# A Novel Serpin with Antithrombin-Like Activity in *Branchiostoma japonicum*: Implications for the Presence of a Primitive Coagulation System

**DOI:** 10.1371/journal.pone.0032392

**Published:** 2012-03-12

**Authors:** Yeqing Chao, Chunxin Fan, Yujun Liang, Bei Gao, Shicui Zhang

**Affiliations:** Department of Marine Biology, Ocean University of China, Qingdao, China; Laboratoire Arago, France

## Abstract

Serine protease inhibitors, or serpins, are a group of widely distributed proteins with similar structures that use conformational change to inhibit proteases. Antithrombin (AT) is a member of the serine protease inhibitor superfamily and a major coagulation inhibitor in all vertebrates, but its evolutionary origin remains elusive. In this study we isolated for the first time a cDNA encoding an antithrombin homolog, *BjATl*, from the protochordate *Branchiostoma japonicum*. The deduced protein BjATl consisted of 338 amino acids sharing 36.7% to 41.1% identity to known vertebrate ATs. BjATl contains a potential N-linked glycosylation site, two potential heparin binding sites and the reactive center loop with the absolutely conserved sequence Gly-Arg-Ser; all of these are features characteristic of ATs. All three phylogenetic trees constructed using Neighbor-Joining, Maximum-Likelihood and Bayesian-Inference methods also placed BjATl together with ATs. Moreover, BjATl expressed in yeast cells was able to inhibit bovine thrombin activity by forming a SDS-stable BjATl-thrombin complex. It also displays a concentration-dependent inhibition of thrombin that is accelerated by heparin. Furthermore, *BjATl* was predominantly expressed in the hepatic caecum and hind-gut, agreeing with the expression pattern of AT in mammalian species. All these data clearly demonstrate that BjATl is an ortholog of vertebrate ATs, suggesting that a primitive coagulation system emerged in the protochordate.

## Introduction

Blood coagulation, or clotting, is of vital importance for the survival of both vertebrates and invertebrates-by preventing the leakage of blood from the sites of injury and impeding infection by the microbial invaders, although the coagulation system of invertebrates is distinct from that of vertebrates [1], [2]. It is known that clotting follows the same fundamental pattern in all vertebrates, culminating the thrombin-catalyzed conversion of fibrinogen to fibrin [3], [4]. How the vertebrate coagulation system evolved from an entirely dissimilar invertebrate coagulation cascade has been a longstanding issue to biologists. Recently, the jawless fish lampreys have been shown to possess a reduced set of clotting factors observed in higher vertebrates [5], while none of the principal clotting factors are found in the urochordate *Ciona intestinalis* [6]. The basal chordate, amphioxus, as the extant representative of subphylum Cephalochordata, has a heart homolog [7] and a circulation system with a fundamental organization found in all chordates [8], [9], providing an ideal model for insights into the origin and evolution of vertebrate coagulation system. Previous studies have shown that amphioxus has plasminogen-like protein [10], [11], [12] and amphioxus humoral fluid has been shown to cross react with human antithrombin antibody [13]. Bioinformatic approaches to inventory the presence or absence of genes involved in blood coagulation processes supports the view that these systems became progressively more complex during the period between the divergence of jawless fish and the appearance of mammals. Furthermore, the root of coagulation systems may extend back to protochordates. However, for this evolutionarily important organism, amphioxus, the coagulation system remains largely unclear.

Serine protease inhibitors, or serpins, are a group of widely distributed proteins with similar structures that use conformational change to inhibit proteases. The first members of the serpin superfamily studied extensively were the human plasma proteins antithrombin and antitrypsin [14], which play key roles in controlling blood coagulation and inflammation. Serpin-like genes have been identified in animals, poxviruses, plants, bacteria and archaea. Eukaryotic serpins have been divided into 16 clades [15]. Antithrombins (also known as antithrombin III; ATs) all belong to the members of the clade C serpin superfamily, which consists of a single chain glycoprotein containing 430 to 439 amino acid residues and has a molecular weight of approximately 58 kDa [16]. There are some common features in clade C members, such as potential N-linked glycosylation site, heparin binding sites and the absolutely conserved reactive center loop sequence Gly-Arg-Ser, which makes them different from other serpin members [16]. AT is able to neutralize most serine proteinases in blood such as thrombin and cofactor Xa by forming stable equimolar complexes with the target enzymes [17], [18], and is thus a major regulator of the blood coagulation system, playing a crucial role in the maintenance of normal hemostasis [19]. The formation of AT-proteinase complexes is slow under physiological conditions, but is accelerated markedly by heparin [20]. AT has been identified in several mammalian species such as humans, cow, horse, pig, sheep, rabbit, mouse, rat and hamster [21]–[25]. It is primarily synthesized in the liver and secreted into plasma [26]–[28], although production by endothelial cells was also reported [29]. AT has also been documented in some non-mammalian vertebrates like cartilaginous fish, bony fish, amphibians, reptiles and birds [30], [16], [31], [32]. So far, ATs have been identified only in vertebrates, and its emergence during animal evolution remains elusive. The purposes of this study was therefore to determine if the *AT-like* gene (designated *BjATl*) is present in the chordate amphioxus *Branchiostoma japonicum*, and if so, to examine its characteristics and expression pattern, and to test if it is functionally similar to vertebrate AT.

## Materials and Methods

### Cloning and sequencing of AT-like cDNA

All animal experiments were carried out in accordance with the guidelines of the Laboratory Animal Administration Law of the People's Republic of China, with the permit number SD2007695 apporved by the ethics committee of the Laboratory Animal Administration of Shandong province.

Total RNAs were extracted with Trizol (Invitrogen) from *B. japonicum* collected in the vicinity of Qingdao, China, and polyA^+^ RNA was purified using polyA tract mRNA isolation system II (Promega) according to the manufacturer's instructions. The first-strand cDNA was synthesized with the reverse transcription system (Promega) using oligo d(T) primer. The fragments of *B. japonicum* AT-like cDNA, *BjATl*, were amplified by PCR with degenerate primer pairs, S1 and A1 (Table 1), which were designed based on the sequences of conserved motifs of vertebrate ATs. The PCR amplification was carried out at 94°C for 3 min, followed by 30 cycles of 94°C for 30 s, 51.6°C for 30 s, 72°C for 90 s and the final extension step at 72°C for 7 min. PCR products were purified and re-amplified. A 988 bp fragment was subcloned and sequenced. The gene-specific primers S2 and A2 (Table 1) were used in RACE (rapid amplification of cDNA ends) reactions for full-length cDNA synthesis, according to the instructions of SMART™ RACE cDNA amplification kit (Clontech.

**Table 1:** Sequences of the primers used in this study.

Primer	Sequence (5′-3′)	Sequence information
S1 (sense)	TCTTCTCWCCBTACAGYATCTC	BjATl cDNA fragment primer
A1 (antisense)	TGDATRAAGAAVAGGAACGG	BjATl cDNA fragment primer
S2 (sense)	CGAAGCTTTGTTGGACGCCACACGAGG	3′RACE primer
A2 (antisense)	TTGCCTCCACCAGTGTGTGCTTGTTC	5′RACE primer
S3 (sense)	CCGGAATTCATGGCCATGACATACATGG	Recombinant primer
A3 (antisense)	CCGCTCGAGATTACTCATTCGGGTTGGTC	Recombinant primer
A4 (antisense)	CTAGTCTAGAGGCTCATTCGGGTTGGTCACC	Recombinant primer

### Sequence analysis

The deduced amino acid sequence was analyzed with the BLAST algorithm at NCBI web site and SWISS-MODEL server at the Expert Protein Analysis System (http://www.expasy.org/). Multiple alignments were performed using ClustalX 1.81 (Thompson et al., 1994) and Multiple Alignment show program (http://www.biosoft.net/sms/index.html). Identity score was obtained using DNAstar software package by Clustal method [33]. Using ClustalX-aligned amino acid sequences, Neighbor-Joining (NJ) tree, Maximum-Likelihood (ML) tree and Bayesian-Inference (BI) tree were constructed. Statistical supports in the NJ tree was represented by percentage of 1000 bootstrap replicates with distances computed by JTT Matrix model in MEGA4.0 [34]. For ML tree, ProtTest 1.4 [35] was used to determine the best protein substitution model and estimate the gamma parameters. After running ProtTest 1.4, the ML tree was constructed using phyML (http://atgc.lirmm.fr/phyml/) by the LG+I+G+F model. In addition, a BI tree was constructed using MrBayes 3.12 [36]. All the sequences used here are listed in Table S1.

The tertiary structure of BjATl was predicted with a homology-modeling method via ESyPred3D using neural networks, using human AT as template [37]. The visualization and characterization of the three-dimensional structures of the human AT and BjATl were performed with software PyMOL [38].

### Preparation of anti-BjATl antibody

The complete coding region of *BjATl* was amplified by PCR with the primer S3 and A3 (Table 1), and sub-cloned into the EcoRI/XhoI site of the pET28a (Novagen) to generate the expression construct pET28a/BjATl with an N-terminal His tag. *Escherichia coli* BL21 transformation and isopropyl β-D-thiogalactoside (IPTG) inducing procedures followed the methods specified by the manufacturer (Novagen). BjATl expressed in *E. coli* was purified using a Ni-NTA resin column (Novagen) according to the manufacturer's protocols. Approximately, 2 mg of the purified BjATl protein was emulsified with Freund's complete adjuvant and injected subcutaneously at multiple sites in rabbits. Three booster injections of 1 mg antigen mixed with Freund's incomplete adjuvant were administered subcutaneously at intervals of 2 weeks. Eight days after the final booster, blood was collected and serum prepared. The antiserum was aliquoted and stored at −70°C until used.

### Expression of BjATl in Pichia pastoris

The complete coding region of BjATl cDNA was amplified by PCR with specific primers S3 and A4 (Table 1). The PCR product was digested with EcoRI and XbaI, and sub-cloned into the plasmid expression vector pPICZαA (Invitrogen) previously cut with the same restriction enzymes. The identity of the insert was verified by sequencing, and the plasmid was designated pPICZαA/BjATl.

The constructed plasmid pPICZαA/BjATl was linearized with SacI and transformed into the competent cells of *P. pastoris* X33 by electroporation as recommended by manufacturer's instructions (Invitrogen). One positive clone was selected and incubated into 100 ml of BMGY medium (1% yeast extract, 2% peptone, 100 mM potassium phosphate, pH 6.0, 1.34% yeast nitrogen base, 4×10^−4^ mg/ml biotin and 1% glycerol) and grown at 28°C until the culture reached OD_600_ = 2–6. The cells were harvested by centrifuging at 2, 000 g for 10 min at room temperature, re-suspended in 500 ml BMMY medium (1% yeast extract, 2% peptone, 100 mM potassium phosphate, pH 6.0, 1.34% yeast nitrogen base, 4×10^−4^ mg/ml biotin and 0.5% methanol) and cultured at 28°C. To induce expression, methanol was added every 24 h to a final concentration of 0.5% for two successive days. The culture was centrifuged at 10, 000 g for 20 min at 4°C. Subsequently, solid (NH_4_)_2_SO_4_ was added to the supernatant to a final concentration of 75% saturation. After stirring at 4°C over night, the suspension was centrifuged at 10, 000 g for 20 min at 4°C. The precipitate was suspended in dialysis buffer (20 mM PBS with 500 mM NaCl, pH 7.4), and dialyzed against the same buffer, which was changed 3 to 4 times, until trace of (NH_4_)_2_SO_4_ was removed. The dialyzed sample was pooled, filtered through a 0.45 µm Millipore filter, and loaded onto a Ni-NTA resin column (Amersham). The column was washed with the washing buffer (20 mM PBS containing 500 mM NaCl and 20 mM imidazole, pH 7.4) and eluted with the elution buffer (20 mM PBS containing 500 mM NaCl and 250 mM imidazole, pH 7.4). The eluted sample was concentrated and solvent exchanged to 50 mM Tris-HCl (pH 7.6) by using Amicon Ultra-15 (MILLPORE). The purity of the recombinant BjATl was analyzed by a 12% SDS-polyacrylamide gel electrophoresis (SDS-PAGE) as described by Laemmli [39], and stained with Coomassie brilliant blue R-250. The recombinant BjATl was aliquoted and stored at −70°C until used. The protein concentrations were determined by the method of Bradford using bovine serum albumin as a standard [40].

### Western blotting

The recombinant BjATl expressed in *P. pastoris* was run on a 12% SDS-PAGE gel under reducing condition. The gel was washed for 15 min in 20 mM PBS containing 0.1% Tween-20, and the proteins on the gel were blotted onto PVDF membrane (Amersham). The blotted membranes were incubated in 20 mM PBS containing 3% defatted milk powder at 30°C for 2 h, and then in the anti-BjATl serum diluted 1∶500 with 20 mM PBS containing 0.1% Tween-20 for 2 h, or in the anti-His antibody (TIANGEN) diluted 1∶1, 000 with the same solution. After washing in 20 mM PBS, the membranes were incubated in horseradish peroxidase (HRP)-labeled goat anti-rabbit IgG (Zhongshan, China) diluted 1∶1,000 at 30°C for 2 h. The bands were visualized using DAB and 0.03% H_2_O_2_.

### Assay for AT-like activity

The activity, if any, of the recombinant BjATl expressed in *P. pastoris* was detected by a chromogenic assay using Actichrome AT III kit (American Diagnostica Inc., Stamford). In this two stage method, 2.5 nkat of bovine thrombin was mixed with 200 µl of 50 mM Tris-HCl (pH 7.6) containing 1.92 µg of BjATl in the presence or absence of 1.8 U/ml heparin. Meanwhile, 2.5 nkat of bovine thrombin was mixed with 200 µl of BjATl solutions that each contained 3.2, 9.6 and 16 µg/ml BjATl, respectively, or with 200 µl of diluted human standard plasma, in the presence of excess of heparin (1.8 U/ml). After initial incubation at 28°C for 30 min, the thrombin-specific chromogenic substrate, Spectrozyme TH, was added to the mixtures, giving a final concentration of 0.18 µM, and incubated at 37°C for 1 min. After addition of 200 µl of acetic acid to terminate the reaction, the residual thrombin activity was determined by measuring the absorbance at 405 nm under a microplate spectrophotometer (GENios Plus Tecan). The inhibitory ability of BjATl on thrombin was inversely proportional to the residual thrombin activity.

### Assay for formation of BjATl-thrombin complex

The purified recombinant BjATl expressed in *P. pastoris* was incubated with bovine thrombin (molecular mass ∼34 kDa) in 50 mM Tris-HCl (pH 7.6) containing excess of heparin (1.8 U/ml), at a molecular ratio 1∶1 at 28°C for 30 min. The reaction products were separated by reducing SDS-PAGE (8%) and immunostained as described above. The humoral fluid was prepared by the method of Wang et al. [41]. Briefly, about 1000 amphioxus were rinsed with distilled water, wiped out thoroughly with sterilized water, and then cut into about 2 mm^3^ pieces on ice to bleed. After centrifugation at 12,000 g at 4°C for 30 min, the supernatant was collected and stored at −70°C until used. Diluted humoral fluids (50 µl; 15 mg proteins/ml) was incubated with bovine thrombin (100 µg) in order to test the presence of native BjATl in *B. japonicum*.

### Northern blotting and In situ hybridization histochemistry

Total RNA was extracted with Trizol (Gibco) from the adult amphioxus *B. japonicum* ground in liquid nitrogen. An aliquot of 5 µg RNAs were each electrophoresed and blotted onto a Nylon membrane (Roche, Germany). The digoxigenin (DIG)-labeled BjATl riboprobes of about 1000 bp were synthesized *in vitro* from linearized plasmid DNA following the DIG-UTP supplier's instructions (Roche, Germany). Northern blot analysis was carried out as described previously [42].

The sexually-matured amphioxus were cut into 3 to 4 pieces and fixed in freshly prepared 4% paraformaldehyde in 100 mM phosphate buffered saline (PBS; pH 7.4) at 4°C for 8 h. The samples were dehydrated, embedded in paraffin, and sectioned at 6 µm. The sections were mounted onto poly-L-lysine coated slides, dried at 42°C for 36 h, and de-paraffinized in xylene for 20 min (two changes for 10 min each), followed by immersion in absolute ethanol for 10 min (two changes for 5 min each). They were re-hydrated, and equilibrated in double distilled H_2_O containing 0.1% DEPC. The digoxigenin (DIG)-labeled BjATl riboprobes of about 500 bp were synthesized *in vitro* from linearized plasmid DNA following the DIG-UTP supplier's instructions (Roche). *In situ* hybridization histochemistry was carried out as described by Fan et al. [42].

## Results

### Sequence and phylogeny of BjATl

A cDNA fragment of approximately 988 bp containing the domain SERPIN was obtained from *B. becheri* by PCR using the degenerate primer pair S1 and A1. The sequences of these primers were designed based on the conserved domain from known antithrombin sequences. Based on the partial cDNA sequence, the primers for 3′RACE and 5′RACE, S2 and A2, were designed, and two cDNA fragments of 1339 bp and 259 bp in length were produced by PCR using S2 and A2, respectively. The full-length cDNA of BjATl was assembled by overlapped cDNA fragments, and was deposited in GenBank (accession number: **EU164803**). The cDNA was 1943 bp long, and included an open reading frame (ORF) of 1017 bp, a 5′-untranslated region (UTR) of 29 bp and a 3′-UTR of 897 bp. The initiation codon, ATG, was in accordance with Kozak rule (A/GXXATGG), and the 3′-UTR had the polyadenylation signal AATAAA. The ORF coded for a deduced protein of 338 amino acids. There was a potential N-linked glycosylation site located at the residual position N33, but it lacked a signal peptide at its N-terminus as predicted by the Signal IP 3.0 server [43].

**Figure 1:**
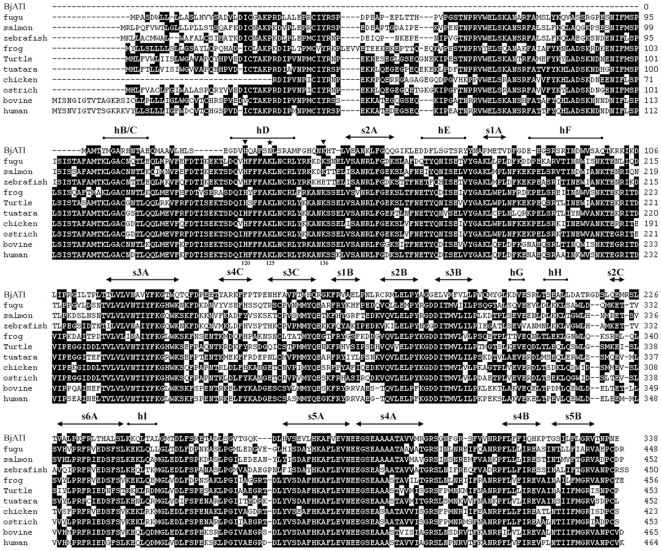
Aligned sequences of BjATl and 10 vertebrate antithrombins. Mature human antithrombin numbering is used. Secondary structural elements of BjATl predicted based on the structure of human antithrombin are shown above the sequences. Solid arrows indicate β-sheet, cylinders represent α-helices, triangles show the heparin-binding sites, and star indicates potential glycosylation site. Amino acid residues that are conserved in at least 50% of sequences are shaded in dark.

**Figure 2:**
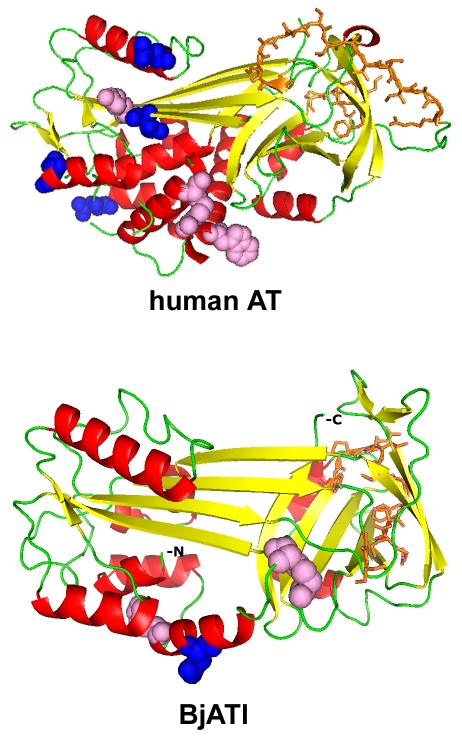
Cartoon representation of homology models of the human AT (A) and BjATl (B). α-helix residues are colored with red, β-sheet residues with yellow, and loop and unassigned residues with green. Pink spheres show the heparin-binding sites, and blue spheres indicate the potential glycosylation site. Orange sticks show the RCL (reactive center loop) region.

Blastp searching at NCBI revealed that BjATl had the conserved domain SERPIN at residues 1–336, and shared 38.2%, 36.7%, 38.5%, 41.1%, 39.1%, 39.6%, 39.6%, 41.7%, 38.5% and 40.8% identity to the antithrombins from fugu, salmon, zebrafish, frog, turtle, tuatara, chicken, ostrich, cow and humans, respectively (Fig. 1). Also, BjATl shared ∼40% indentity with some serpin clade B members, such as Bovine SCCA (XP001254097), Bovine PI-6 (O02739) and Human SCCA (P29508). The predicted 3D structures of human AT and BjATl are shown in Figure 2. Although the numbers of β-sheets at N-termini (BjATl had 3 β-sheets, while human AT had 6 β-sheets) and glycosylation sites in human AT and BjATl were different, their general 3D structures show significant similarity. Moreover, the reactive side loop region of BjATl was closely resembles that of human AT.

**Figure 3:**
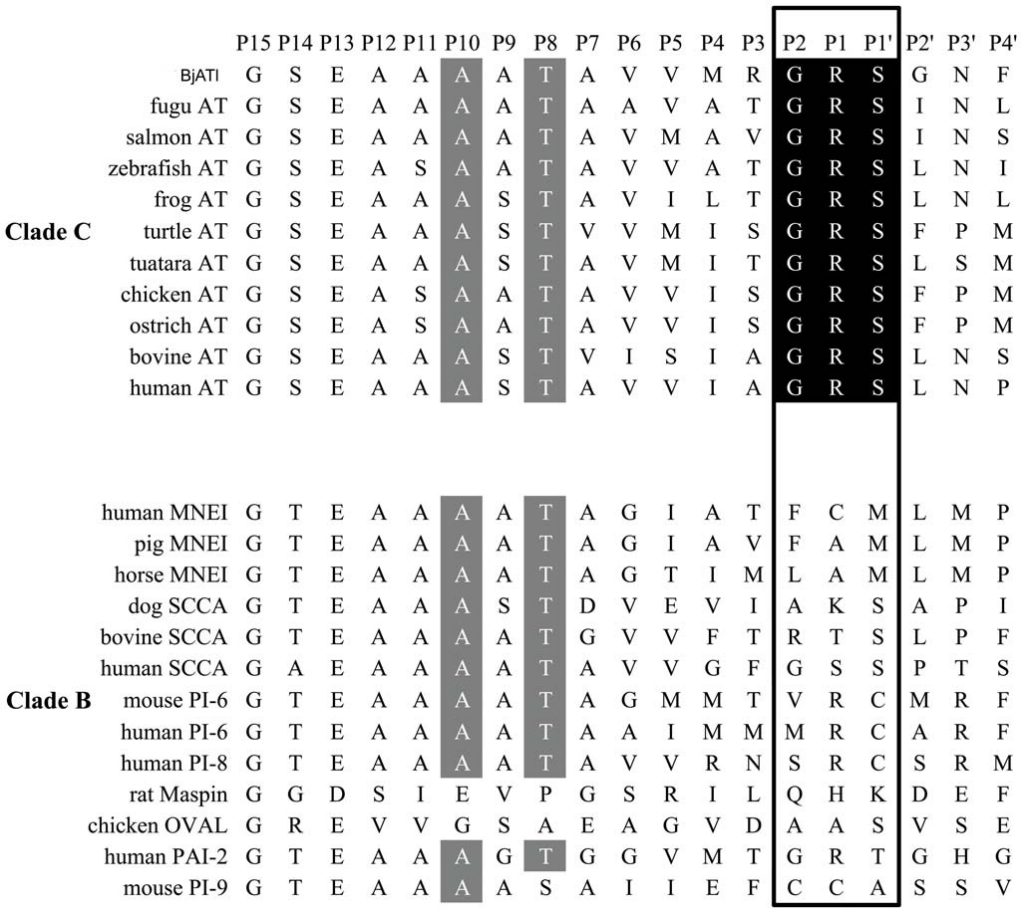
Comparison of serpin RCLs. Clade C (upper panel) and Clade B (lower panel) serpin RCLs from P15-P4′ were aligned. Residues from P2, P1 and P1′ are framed as box, and the residues absolutely conserved are shaded in dark. The strictly conserved residues at P8 and P10 are shaded in grey.

Sequence comparison showed that BjATl contains a reactive center loop (RCL) similar to that of ATs. The RCL forms an extended and exposed conformation above the body of AT scaffold, and is responsible for the interaction with target proteases. The 20 amino acid residues constituting the RCL are numbered Pn- … -P1-P1′- … -Pn′, where P1-P1′ is ultimately cleaved [44]. The residues P2, P1 and P1′ with the sequence Gly-Arg-Ser, the primary determinants of AT specificity, were absolutely conserved in BjATl and other ATs (Fig. 3). Besides, the P8 (Thr) and P10 (Ala), which are important for the formation of covalent complex with target proteinase, were also strictly conserved in BjATl and other ATs. Comparisons to human antithrombin shows BjATl contains the potential heparin binding site residues H120 and K136 (numbering as human AT; [16]) although it did not contain the heparin binding site residues K11, R13, R46, R47, K125, R129, R132 and K133. Interestingly, in BjATl the K125 is replaced by asparagine (an N-linked glycosylation site) (Fig. 1), which may also play a crucial role in heparin binding [45].

**Figure 4:**
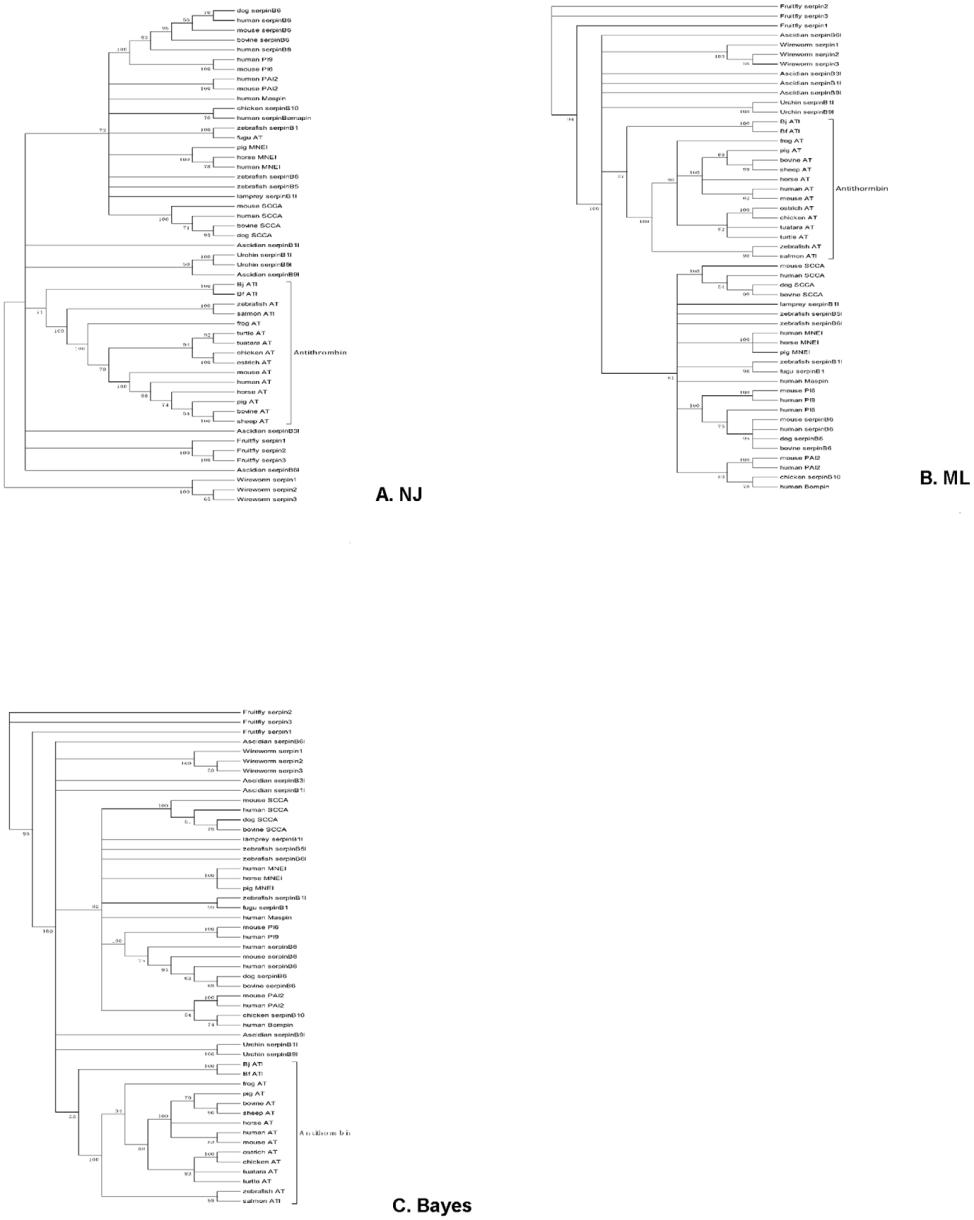
The phylogenetic trees constructed using the sequences of BjATl and other representative members of serpin cladeB and cladeC. (A) The neighbor-joining (NJ) tree constructed using the package MEGA4.0; (B) The maximum likelihood (ML) tree using the program PhyML3.0; and (C) The Bayesian inference (BI) tree using MrBayes3.04b. Branches with bootstrap value <50% are cut off. Accession numbers for the sequences used are listed in Table S1.

Among the 16 clade serpins, BjATl shared high sequence identity with clade B and clade C serpins. The clade B serpins lack the signal peptide, are primarily intracellularly localized, and are supposedly the ancestors to the majority of extracellular serpin proteins (including ATs) [46]. Like clade B members, BjATl does not have signal peptide. In contrast, the residues at P2, P1 and P1′ of BjATl are different from clade B members; they are Gly-Arg-Ser, which are absolutely conserved in and typical of ATs (Fig. 3). Both clade B and clade C serpins were included in the phylogenetic tree construction. As shown in Figure 4, all the phylogenetic trees constructed by different methods revealed that BjATl was clustered together with ATs, and located at the root of antithrombin (clade C serpin) branch, separating from clade B serpin members. These indicated that BjATl is an ortholog of antithrombins (clade C serpin).

### Expression of BjATl in yeast cells

**Figure 5:**
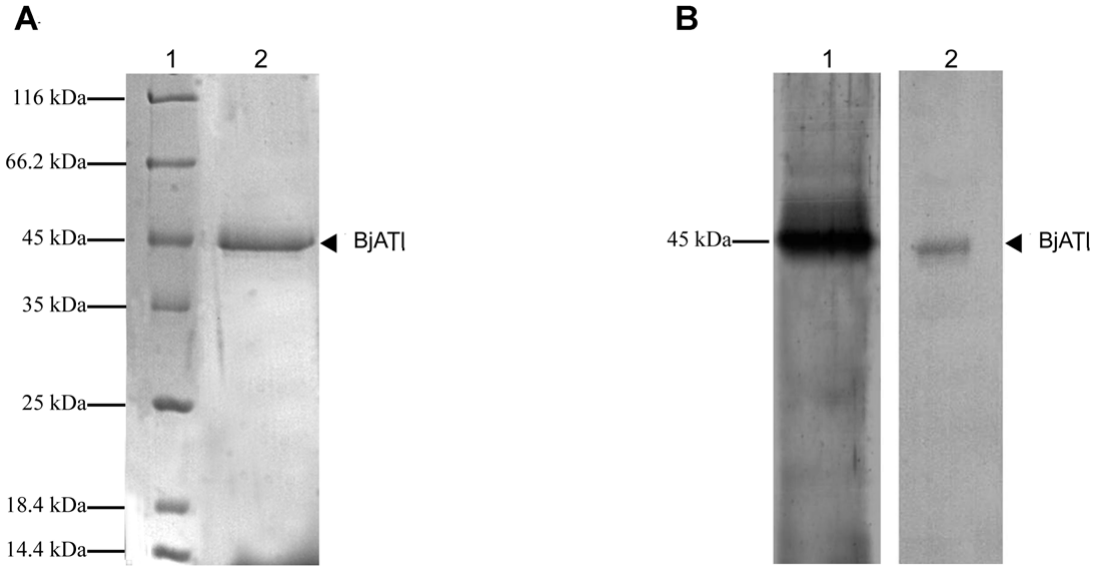
SDS-PAGE and Western bloting of recombinant BjATl expressed in *Pichia pastoris*. (A) SDS-PAGE of recombinant BjATl purified on Ni-NTA resin column. Lane 1, molecular mass standards; Lane 2, recombinant BjATl. (B) Western blotting. Lane 1, the supernant of *Pichia pastoris* with *BjATl* insertion induced with methanol, and immunostained with anti-BjATl antiserum; Lane 2, the supernant of *Pichia pastoris* with *BjATl* insertion induced with methanol, and immunostained with anti-His tag antiserum.

The constructed plasmid pPICZαA/BjATl was linearized with SacI and transformed into *P. pastoris* X33. The positive clones were screened and utilized for expression. The recombinant protein with the His-tag was purified by affinity chromatography on a Ni-NTA resin column, and analyzed by a 12% SDS-PAGE, followed by staining with Coomassie Brilliant Blue R-250, which demonstrated the presence of a single protein band of approximately 45 kDa (Fig. 5A). Western blotting revealed that the purified protein reacted with both rabbit anti-BjATl serum and anti-His-tag antibody, indicating that BjATl was correctly expressed (Fig. 5B).

### Inhibitory effect of BjATl on bovine thrombin activity

**Figure 6:**
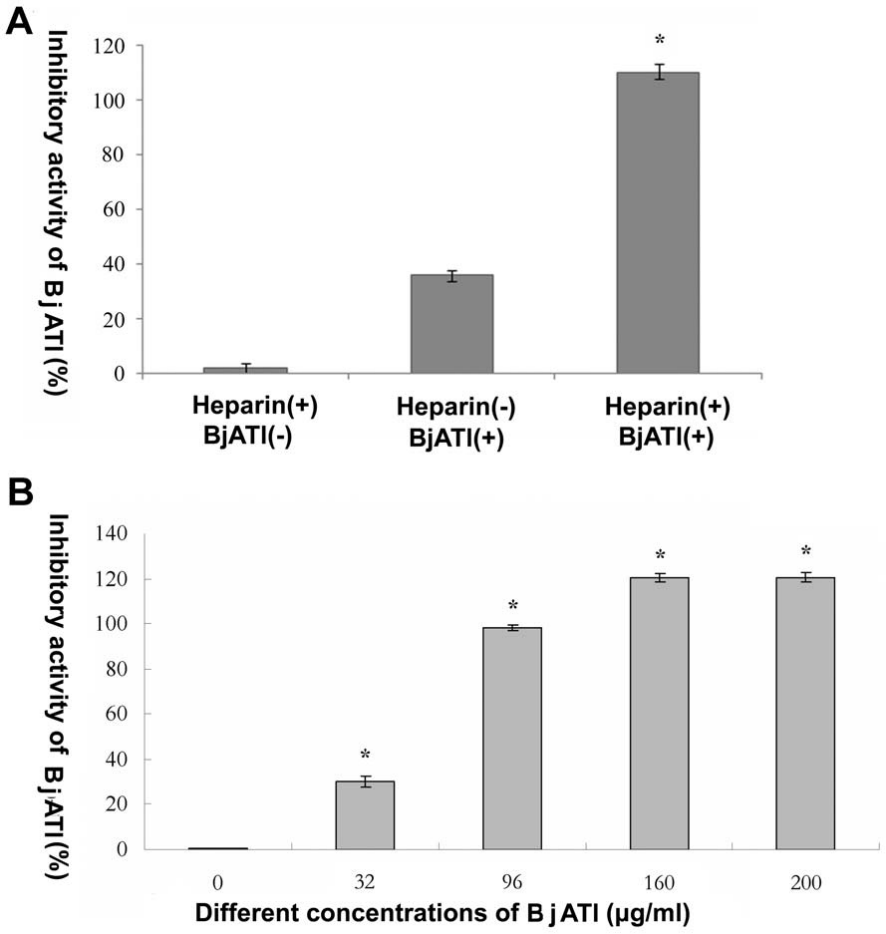
Inhibitory activity of recombinant BjATl. (A) The inhibitory activity of recombinant BjATl in the presence (+) or absence (−) of heparin. (B) The inhibitory activity of different concentrations of recombinant BjATl in the presence of heparin. The inhibitory activity of BjATl was determined for each group and values were shown as means ± SD (n = 3). Significant differences (*p*<0.001) are indicated by an asterisk (*).

The inhibitory activity of BjATl was quantified by comparison to a standard curve prepared with diluted normal human plasma. By definition, AT activity of diluted normal plasma is 100%. As shown in Figure 6, BjATl was capable of inhibiting bovine thrombin activity in a concentration-dependent manner, and its inhibitory activity was significantly accelerated by heparin.

### BjATl forms SDS-stable complex with thrombin

**Figure 7:**
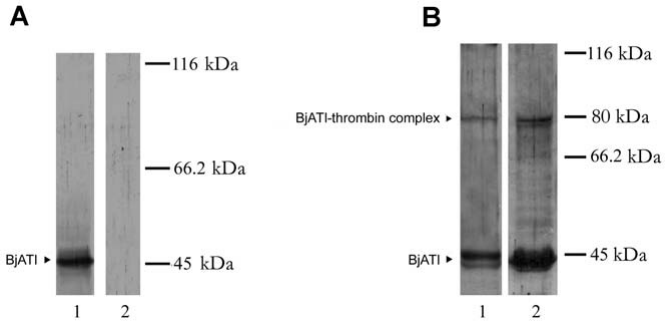
Analysis of complex formation with thrombin. Purified BjATl or amphioxus humoral fluids were incubated with bovine thrombin. After SDS-PAGE (8% gels) under reducing condition, the reaction products were immunostained with anti-BjATl antiserum. (A) Lane 1, purified BjATl; Lane 2, bovine thrombin. (B) Lane 1, purified BjATl incubated with bovine thrombin; Lane 2, amphioxus humoral fluids incubated with bovine thrombin. The positions and molecular masses of marker proteins are indicated on the right.

To detect the interaction between BjATl and thrombin, BjATl was exposed to bovine thrombin. Pilot experiments showed that anti-BjATl serum reacted with BjATl, forming a single band of ∼45 kDa, whereas it was not reactive with bovine thrombin (Fig. 7A). Western blotting revealed that the incubation of bovine thrombin with recombinant BjATl resulted in the formation of a SDS-stable complex (Fig. 7B), which had a molecular mass of ∼80 kDa (BjATl-thrombin complex). Another protein band was observed to migrate slightly faster than the residual non-reacted BjATl, which is apparently the cleaved BjATl as reported by Mochizuki et al [47]. Similarly, the incubation of bovine thrombin with *B.japonicum* humoral fluids led to the occurrence of two major bands at ∼45 kDa and ∼80 kDa (Fig. 7B), suggesting the presence of native BjATl protein in *B. japonicum*, which can interact with thrombin.

### Tissue-specific expression of BjATl in adult amphioxus

**Figure 8:**
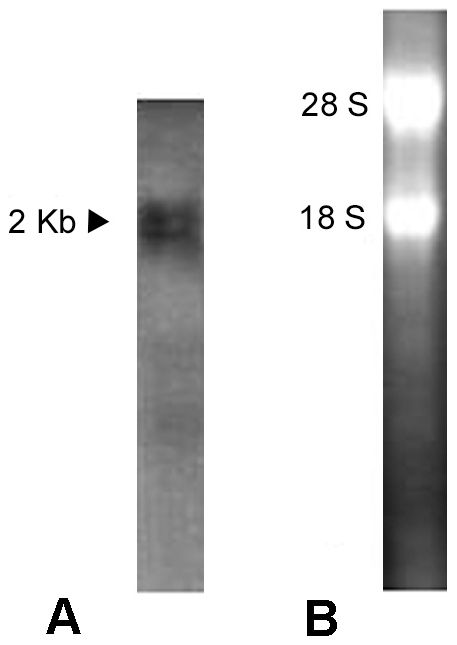
Northern blotting. (A) The blot was hybridized with Dig-labeled BjATl RNA probe. The arrow indicates the position of molecular size equivalent to 2000 bp. (B) A total of 5 µg RNA was analyzed in 1.2% agarose formaldehyde-denaturing gel.

**Figure 9:**
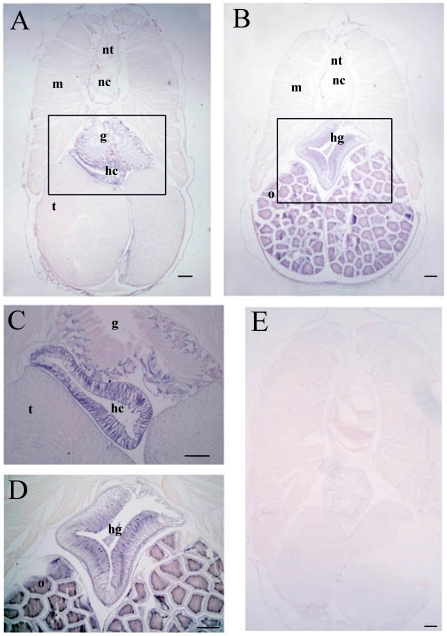
Localization of *BjATl* transcripts in different tissues of adult amphioxus detected by *in situ* hybridization histochemistry. (A) A low magnification section of a male amphioxus showing the presence of *BjATl* mRNA was most abundant in hepatic caecum (hc) and at a lower level present in gill (g). No signal was found in testis (t), muscle (m), notochord (nc) and neural tube (nt). (B) A low magnification section of a female amphioxus showing the presence of *BjATl* mRNA was most abundant in hind-gut (hg) and at a lower level present in ovary (o). (C) and (D) The enlargement of the boxs in A and B. (E) Micrograph showing the absence of *BjATl* transcripts in control section. Scale bars represent 100 µm.

Northern blotting revealed the presence of an approximately 2000 bp transcript in *B. japonicum* (Fig. 8). To explore the expression pattern of *BjATl* in adult *B. japonicum*, tissue section *in situ* hybridization was conducted and the results demonstrated that *BjATl* transcript was most abundant in the hepatic caecum and hind-gut, and at a lower level present in the gill and ovary, while it was absent in the epidermis, muscle, neural tube, notochord and testis (Fig. 9), implicating a tissue-specific expression pattern of *BjATl* in adult *B. japonicum*.

## Discussion

Previous studies have shown the presence of AT in jawed vertebrates [1], while it was recently found that a putative AT-like homolog is present in amphioxus *B. japonicum* [12]. Here we demonstrate for the first time a novel member of serpin family with AT-like activity in *B. japonicum*. The deduced 338 amino acids long protein, BjATl, shares more than 36.7% identity to known ATs and contains the conserved domain SERPIN at residues 1–336 (including the RCL with the conserved AT specific sequence GRS), an N-linked glycosylation site and the potential two heparin binding sites. Additionally, the recombinant BjATl exhibits thrombin-inhibiting activity, which can be enhanced by heparin. Mammalian antithrombin inactivates the coagulation protease thrombin by forming stable equimolar AT/target enzyme complex [48], [49]. BjATl is also able to interact with bovine thrombin in the presence of heparin by forming BjATl-thrombin complex (Fig. 7B), suggesting that BjATl, like mammalian AT, utilizes a similar mechanism to bind to thrombin. Both sequencing and functional data clearly indicate that BjATl is a novel member of serpin with some AT-like activity. Previously, plasminogen-like protein has been identified in amphioxus [13]. Taken together, these findings appear to provide us a clue that a primitive coagulation system already emerged in the protochordate.

Clade B serpins lack signal peptide and reside primarily within cells, most members are normally shorter (350–400 amino acides [50]) than ATs. These Clade B serpins are presumed to be ancestors of the majority of extracellular serpins (including antithrombins) [46]. It is of interest to note that BjATl shares ∼40% identity with some clade B members. Also, all the three phylogenetic trees show that BjATl groups at the root of clade C (ATs) branch. It is likely that BjATl is the common ancestor of clade B and clade C serpins. These members of the serpin family currently present in mammals, avians and amphibians may have evolved through intragenic duplication and N-terminal amino acid replacement of the protease domain, gene duplication, and exon shuffling and deletion.

Several clade B serpins were found to exist in both intracellular and extracellular forms [46], [51]. Western blotting results reveal that BjATl is secreted and circulates in the humoral fluids at low levels. This also suggests that the molecular weight of native BjATl is approximately 45 kDa, which is closely similar to recombinant BjATl. As the recombinant BjATl used here is expressed in *P. pastoris* X33, and this eukaryotic expression system has the advantage that allows protein glycosylation to take place, it is therefore possible that the function of recombinant BjATl is a partial reflection to native BjATl. It is of note that the molecular mass of BjATl is smaller than that estimated from Liang's study [12]. The reason for this difference is not clear at present, and needs to be clarified in the future.

The liver is the major synthesis site of AT in vertebrates [27]–[29]. Amphioxus has a hepatic caecum, the pouch that protrudes forward as an outpocketing of the digestive tube and extends along the right side of the posterior part of the pharynx, which has long been considered to be the homologous structure to vertebrate liver [52]–[54]. Our study reveals that BjATl exhibits a tissue-specific expression pattern in *B. japonicum*, with the most abundant expression in the hepatic caecum and hind-gut. Broadly speaking, this supports that the homology of the hepatic caecum of amphioxus to the vertebrate liver.

In summary, the present study demonstrates molecularly and functionally the presence of a novel member of serpins with AT-like activity in amphioxus *B. japonicum*, pushing the evolutionary origin of this protein to the invertebrate chordate. This suggests that a pritimitive coagulation system already emerged in the protochordate.

## Supporting Information

Table S1 **The names and accession numbers of serpins.**
(DOC)
