# Supplementary material for: A Novel Serpin with Antithrombin-Like Activity in Branchiostoma japonicum: Implications for the Presence of a Primitive Coagulation System
Source: PLoS One. 2012 Mar 12;7(3):e32392. doi: 10.1371/journal.pone.0032392 (PMC3299649; doi:10.1371/journal.pone.0032392)
Supplement: Table S1 — The names and accession numbers of serpins. (DOC) [file pone.0032392.s001.doc]

Table 2. The names and accession numbers of serpins

|  | **Species** | **Name** | | **Accession numbers** |
| --- | --- | --- | --- | --- |
|  | *Branchiostoma belcheri* | | BjATl | **ABW74215** |
|  | *Xenopus laevis* | | Frog AT | **NP_001080079** |
|  | *Chelydra serpentina* | | Turtle AT | **AAL60466** |
|  | *Sphenodon punctatus* | | Tuatara AT | **AAL73207** |
|  | *Gallus gallus* | | Chicken AT | **AAB35653** |
|  | *Struthio camelus* | | Ostrich AT | **AAL60465** |
|  | *Mus musculus* | | Mouse AT | **AAB23965** |
|  | *Homo sapiens* | | Human AT | **NP_000479** |
|  | *Equus caballus* | | Hourse AT | **XP_001497366** |
|  | *Sus scrofa* | | Pig AT | **ABF82362** |
|  | *Bos taurus* | | Bovine AT | **NP_001029870** |
|  | *Ovis aries* | | Sheep AT | **NP_001009393** |
|  | *Danio rerio* | | Zebrafish AT | **AAN71002** |
|  | *Salmo salar* | | Salmon ATl | **CAB64714** |
|  | *Gallus gallus* | | Chicken OVAL | **P01012** |
|  | *Coturnix japonica* | | Japanese quail OVAL | **P19104** |
|  | *Meleagris gallopavo* | | Turkey OVAL | **AAC16664** |
|  | *Homo sapiens* | | Human serpinB6 | **P35237** |
|  | *Homo sapiens* | | Human serpinB8 | **P50452** |
|  | *Mus musculus* | | Mouse PI9 | **AAB57812** |
|  | *Homo sapiens* | | Human PI9 | **P50453** |
|  | *Gallus gallus* | | Chicken MENT | **AAC15710** |
| *Homo sapiens* | | | Human Bomapin | **P48595** |
| *Mus musculus* | | | Mouse PAI2 | **P12388** |
| *Homo sapiens* | | | Human PAI2 | **P05120** |
| *Equus caballus* | | | Horse MNEI | **P05619** |
| *Homo sapiens* | | | Human MNEI | **P30740** |
| *Sus scrofa* | | | Pig MNEI | **P80229** |
| *Homo sapiens* | | | Human Maspin | **P36952** |
| *Mus musculus* | | | Mouse PI6 | **Q60854** |
| *Bos taurus* | | | Bovine SCCA | **XP_001254097** |
| *Canis familiaris* | | | Dog SCCA | **XP_541074** |
| *Homo sapiens* | | | Human SCCA | **P29508** |
| *Bos taurus* | | | Bovine SerpinB6 | **O02739** |
| *Canis familiaris* | | | Dog SerpinB6 | **XP_848373** |
| *Rattus norvegicus* | | | Rat Maspin | **P70564** |
| *Mus musculus* | | | Mouse SCCA | **NP_033152** |

| *Danio rerio* | Zebrafish serpinB1 | **NP_001002653.1** |
| --- | --- | --- |
| *Danio rerio* | Zebrafish serpinB5 | **NP_998042.1** |
| *Danio rerio* | Zebrafish serpinB6 | **NP_001103200.1** |
| *Tetraodon nigroviridis* | Fugu serpinB1 | **CAF98097.1** |
| *Petromyzon marinus* | Lamprey serpinB1l | **ENSPMAP00000009944** |
| *Ciona intestinalis* | Ascidian serpinB1l | **XP_002119928.1** |
| *Ciona intestinalis* | Ascidian serpinB3l | **XP_002130310.1** |
| *Ciona intestinalis* | Ascidian serpinB6l | **XP_002128201.1** |
| *Ciona intestinalis* | Ascidian serpinB9l | **XP_002125924.1** |
| *Strongylocentrotus purpuratus* | Urchin serpinB1l | **XP_786830.2** |
| *Strongylocentrotus purpuratus* | Urchin serpinB9l | **XP_790133.1** |
| *Caenorhabditis elegans* | Wireworm serpin1 | **AAS13527.1** |
| *Caenorhabditis elegans* | Wireworm serpin2 | **AAB71270.1** |
| *Caenorhabditis elegans* | Wireworm serpin3 | **AAS13529.1** |
| *Drosophila melanogaster* | Fruitfly serpin1 | **AAF57408.2** |
| *Drosophila melanogaster* | Fruitfly serpin2 | **AAF52627.2** |
| *Drosophila melanogaster* | Fruitfly serpin3 | **AAF53932.3** |
| *Branchiostoma floridae* | BfATl | **XP_002600141.1** |
